# Viability of Bacteriophages in the Complex Hydrogel Wound Dressings *in vitro*

**DOI:** 10.17691/stm2021.13.2.03

**Published:** 2021-04-30

**Authors:** V.V. Beschastnov, M.G. Ryabkov, A.E. Leontiev, A.A. Tulupov, T.N. Yudanova, I.Yu. Shirokova, N.А. Belyanina, O.V. Kovalishena

**Affiliations:** Associate Professor, Senior Researcher, University Clinic, Privolzhsky Research Medical University, 10/1 Minin and Pozharsky Square, Nizhny Novgorod, 603005, Russia; Associate Professor, Leading Researcher, University Clinic, Privolzhsky Research Medical University, 10/1 Minin and Pozharsky Square, Nizhny Novgorod, 603005, Russia; Associate Professor, Senior Researcher, University Clinic, Privolzhsky Research Medical University, 10/1 Minin and Pozharsky Square, Nizhny Novgorod, 603005, Russia; Junior Researcher, University Clinic, Privolzhsky Research Medical University, 10/1 Minin and Pozharsky Square, Nizhny Novgorod, 603005, Russia; Head of the Laboratory, LLC “New Dressing Materials”, 2i Sergiev Posad District, Zhuchki, Moscow Region, 141351, Russia; Head of the Laboratory Research Department, University Clinic, Privolzhsky Research Medical University, 10/1 Minin and Pozharsky Square, Nizhny Novgorod, 603005, Russia; Junior Researcher, University Clinic, Privolzhsky Research Medical University, 10/1 Minin and Pozharsky Square, Nizhny Novgorod, 603005, Russia; Professor, Head of the Department of Epidemiology, Microbiology and Evidence-Based Medicine, Privolzhsky Research Medical University, 10/1 Minin and Pozharsky Square, Nizhny Novgorod, 603005, Russia

**Keywords:** bacteriophages, hydrogel, wound dressing, succinic acid, lidocaine

## Abstract

Using bacteriophages to overcome the increasing resistance of microorganisms to antibiotics is a novel research venue of clinical importance. Among other challenges, this technique is expected to create and maintain an adequate local concentration of bacteriophages at the site of application. In addition, the possibility of combining the phage preparation with antioxidants and anesthetics may provide new options for stimulating the reparative process.

**The aim of the study** was to assess the viability and lytic activity of bacteriophages incorporated into a hydrogel-based wound dressing that contains polyvinyl alcohol, phosphate buffer, with optional additions of succinic acid and lidocaine.

**Materials and Methods:**

A technique for incorporating bacteriophages into the complex hydrogel wound dressing *ex tempore* has been proposed. The bacteriolytic activity of phages inside the hydrogel was determined using standard microbiological techniques. Specifically, we used nutrient media with lawn cultures of *Staphylococcus aureus* added with the following antibacterial combinations: bacteriophages + succinic acid, bacteriophages + lidocaine, and bacteriophages + succinic acid + lidocaine. The lytic activity of bacteriophages was assessed within 1 to 7 days after the formation of the hydrogel.

**Results:**

In all samples containing bacteriophages, the presence of viable and lytically active phages was noted within 1 to 7 days, as evidenced by the “negative colonies” on the culture lawns. On days 1 to 3, no secondary growth was recorded in the phage-containing samples. In hydrogel samples containing phages, succinic acid, and lidocaine, secondary bacterial colonies were detected starting from day 4 indicating some reduction in the lytic activity.

**Conclusion:**

The results suggest that bacteriophages immobilized in the hydrogel maintain their viability and lytic activity, and this activity persists when the phages are combined with succinic acid and lidocaine.

## Introduction

Although the apocalyptic claims about the “end of the antibiotic era” may be considered an exaggeration, it is already clear that the multiple antibiotic resistance of microorganisms is becoming a clinical, epidemiological, and economic problem. One of the promising and rapidly developing approaches to overcome the increasing antibiotic resistance is the use of bacteriophages, i.e. viruses that infect bacteria and serve as natural regulators of bacterial multiplication [1–3]. In particular, a lot of attention is paid to the fight against hospital wound infections, which, on the one hand, aggravate the patient’s condition, and on the other hand, complicate surgical interventions aimed to close the skin defect.

The use of bacteriophages against pathogenic microorganisms attracts the attention of surgeons around the world. Along with that, experts in free skin grafting suggest that the infection control should be combined with support of redox reactions and angiogenesis in the skin around the wound in order to improve the engraftment of an autodermotransplant [4]. It was proposed that succinic acid [5, 6], and lidocaine (an anesthetic) [7] could support the activity of redox reactions and angiogenesis when used as components of hydrogel wound dressings. However, to date, no studies reported on the viability of such unique biological preparations as bacteriophages combined with hydrogel wound dressings and added with succinic acid and lidocaine.

**The aim of the study** was to assess the viability and lytic activity of bacteriophages incorporated into a hydrogel-based wound dressing that contains polyvinyl alcohol, phosphate buffer, with optional additions of succinic acid and lidocaine.

## Materials and Methods

The studies were carried out in the microbiological laboratory of the University Clinic of the Privolzhsky Research Medical University (Nizhny Novgorod, Russia). Experimental wound dressing samples were provided by the LLC “New Dressing Materials” (Moscow, Russia).

To model a wound dressing, we used a film made of polyvinyl alcohol (PVA) and phosphate buffer (PB) with pH 6.6–7.8, at a concentration of (1–3)·10^–5^ mol/g. This composition was capable of converting into a gel and incorporating 0.05–0.20 ml/cm^2^ of a bacteriophage mixture. In this preparation, PVA — a hydrophilic biocompatible polymer — serves as a suitable matrix for the immobilization of bioactive substances [5, 8]. The role of PB as a component of the film is to create an optimal pH for maintaining the viability of bacteriophages.

To assess the bacteriolytic activity of bacteriophages in this composition, four samples of wound dressing were examined in two series of experiments (Table 1). When mixed with a solution of bacteriophages, the film is converted into a gel within 30–60 s; consequently, a gel plate is formed and the film swells by 500 to 1200%. Sample 1 contained the main components of the dressing — PVA and PB at a concentration of (1–3)·10^–5^ mol/g (dry matter); sample 2 contained lidocaine (in addition to PVA and PB) at a concentration of 4.2 mg/g PVA. In samples 1 and 2, the pH was maintained at 7.6. Samples 3 and 4 had a pH of 7.2, since sample 3 contained succinic acid at 0.26 mg/g PVA, and sample 4 — both succinic acid (0.26 mg/g PVA) and lidocaine (4.2 mg/g PVA).

**Table 1:** Composition of hydrogel wound dressing samples

Samples	рН	Concentration in dry film (mg/g PVA)	
		Succinic acid	Lidocaine
1	7.6	0	0
2	7.6	0	4.2
3	7.2	0.26	0
4	7.2	0.26	4.2

The film thickness was 90±10 μm. It was packed in individual bags (air- and waterproof) that were sterilized by radiation at a dose of 20±5 kGy.

For the study, we used an aqueous solution of bacteriophages “Complex Pyobacteriophage” (Microgen, Russia). According to the manufacturer’s description, this product is able to lyse the following microorganisms: staphylococci, streptococci, enterococci, Proteus spp. (*P. vulgaris*, *P. mirabilis*), Klebsiella spp. (*K. pneumoniae*, *K. oxytoca*), as well as *Pseudomonas aeruginosa* and *Escherichia coli*.

To determine the viability and lytic activity of the phages, we followed the federal clinical guidelines [9] that included:

preparation of nutrient media;

preparation of bacterial suspensions;

sowing of bacteria on nutrient media;

preparation of various modifications of a hydrogel by adding bacteriophages to the polymer film;

placement of samples on lawns of bacterial cultures of the strains tested;

incubation;

quantification and interpretation of the results, drawing conclusions on the viability and lytic activity of the phages.

In this study, we used standard nutrient media, on which the tested microorganisms provided a visible growth under appropriate conditions. The culture medium was prepared from a commercially available dry powder in accordance with the manufacturer’s instructions. After autoclaving, the medium was poured into sterile Petri dishes to produce a layer not deeper than 4 mm. The dishes were left at room temperature for solidification and drying needed for further phage lysate absorption and condensate evaporation. A bacterial culture was applied to the dry surface of the nutrient medium with a bacteriological loop. The concentration of microorganisms in the inoculum was 1.5·10^8^ CFU/ml. The inoculum was prepared in accordance with the methodological instructions MUK 4.12.1890-04.

To simulate the wound dressing samples, each film was cut into square pieces of 1×1 cm using sterile scissors; these pieces were then placed in Petri dishes and poured with aliquots of the bacteriophage solution. After 60 s, the film got converted into a gel. The viability, bioavailability (release), and lytic activity of the phages were determined using lawns of the bacterial cultures.

The pandrug-resistant *Staphylococcus aureus* strain obtained from a patient in the local burn center was used as a test strain. The culture was plated as a lawn throughout the entire Petri dish. A few minutes after the culture turned solid, specimens of the tested material were applied to the surface of each plate. After the samples got attached to the agar surface, the plates were turned upside down and placed into a thermostat at 32°C.

To calibrate the measurements of bacteriolytic activity, a drop of the bacteriophage solution was applied on a Petri dish with a lawn of *Staphylococcus aureus* (control 1). The second control sample contained a prototype wound coating prepared by adding saline to the film (control 2). We also tested another prototype obtained by applying the bacteriophage solution to the film (control 3). All three controls were then incubated at 32°C (see the Figure).

**Figure:**
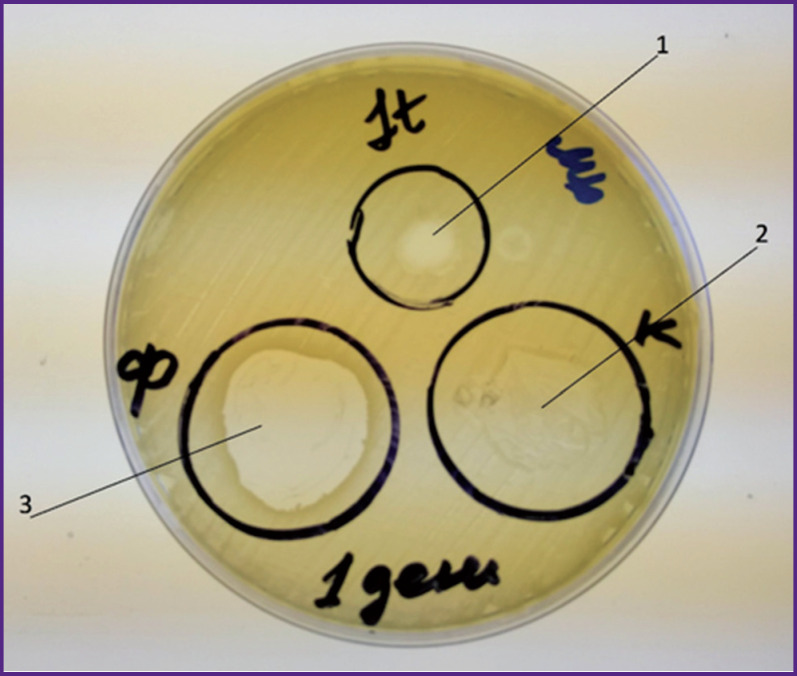
A Petri dish with negative colonies (lysis zones) on lawn cultures of *Staphylococcus aureus* in the presence of: (1) fresh bacteriophage-containing solution (control 1); (2) gel obtained from 0.9% sodium chloride solution (control 2); (3) gel obtained from bacteriophage solution (control 3)

In our experimental set-up, physiological saline (free of bacteriophages) was added to the film in all samples under study. Then, microbiological tests were run to detect a possible lytic activity associated solely with the materials used and not related to bacteriophages themselves.

To determine the viability of bacteriophages in the gel, gel samples with an area of 1 cm^2^ were applied on the lawns of test cultures immediately after the gel plate formation (day 0), and then on days 1, 2, 3, 4, and 7. Quantification and interpretation of the results were done after 24 h. The presence of viable phages in the test medium was confirmed by the appearance of a “sterile spot” at the point where the sample drop was applied. The lysis reactions (complete suppression of visible bacterial growth) were assessed by visual observation under illumination at 45°. As listed below, the results were graded in proportion to the bacteria lysis in the presence of a specific bacteriophage [9]:

1. the test material contains viable and lytically active bacteriophages if a continuous negative phage colony is observed at the site where the test material has been added;
2. the test material contains viable, but lytically weak bacteriophages if, at the site of its application, there are a few negative bacterial colonies or isolated bacterial colonies;
3. the test material does not contain active bacteriophages — the complete absence of any lysis.

The lytic activity of the phage was assessed with a five-point scale (by the number of crosses):

“–” lack of lytic activity; “+” low activity;

“++” formation of a lysis zone with a large number of colonies of secondary bacterial growth;

“+++” a lysis zone with isolated colonies of secondary growth;

“++++” a clear zone of lysis without colonies of secondary growth.

## Results

During the calibration phase, we noticed a number of negative colonies (lysis zones) on the lawns with *Staphylococcus aureus* in the spots where a bacteriophage containing solution (control 1) or a gel (control 3) were added. In the area with a gel produced from 0.9% sodium chloride (control 2), a continuous negative colony was observed, which indicated the lack of lytic activity of the substrate containing a gel based on PVA and PB, succinic acid and lidocaine in relation to the test culture used.

In all control samples, i.e. when saline only was added to the polymer films, areas of negative colonies were not observed, the growth of the culture under the gel plate was preserved (lytic activity was graded as “–”).

In all phage-containing samples (Table 2), the presence of viable and lytically active bacteriophages was noted on day 0, i.e. immediately after the formation of the hydrogel plate. In the area of the hydrogel plate and around it, negative colonies of the test culture were observed; therefore, the lytic activity in these samples was marked as “++++”.

**Table 2:** Petri dishes with lawns of *Staphylococcus aureus* culture and antibacterial hydrogel samples containing various drugs

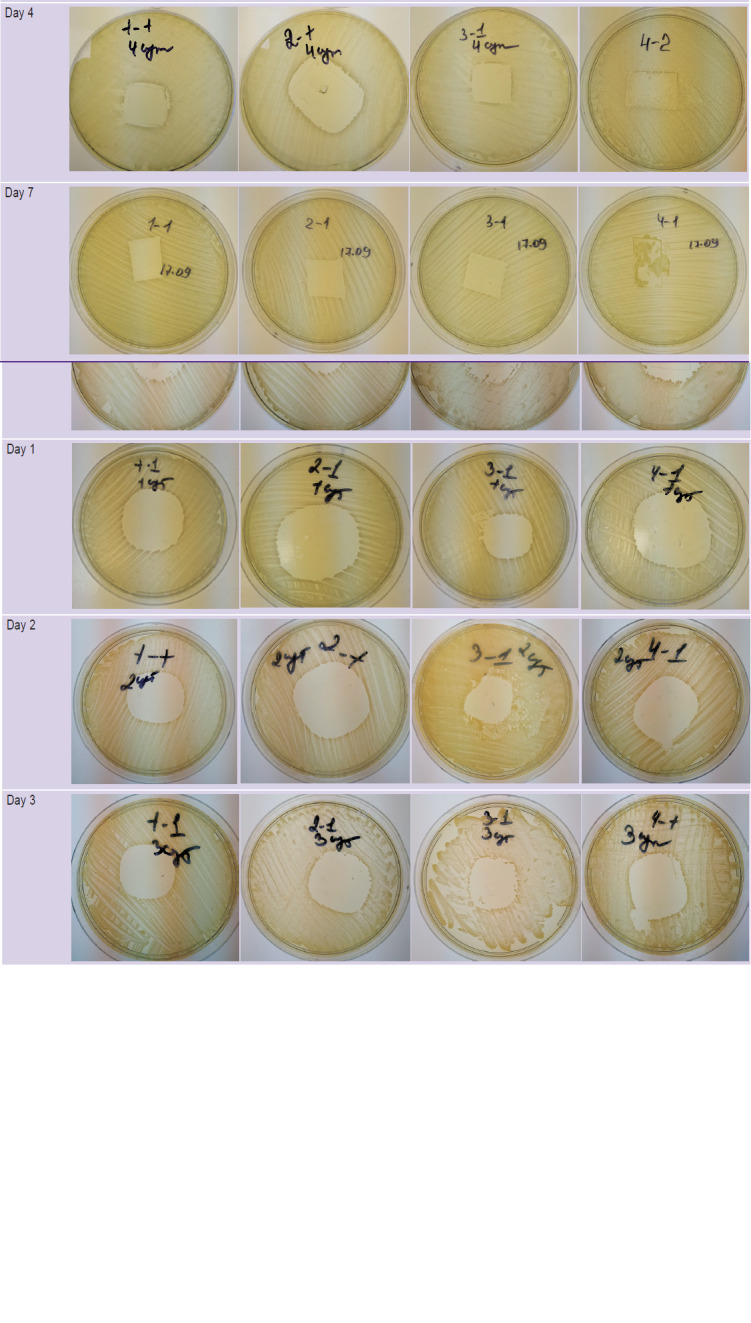

One day after the hydrogel formation, clear lysis zones without signs of secondary growth were detected around the samples placed on the test culture; these zones indicated the presence of viable bacteriophages that preserved their lytic activity in the hydrogel. A slightly smaller diameter of the lysis zone was noted in sample 3 in comparison with other samples. On days 3 and 4, this trend persisted. On day 4, in the 1^st^ series of sample 4, a lysis zone with isolated secondary colonies was noted; there the lytic activity was assessed as “+++”. In the 2^nd^ series of sample 4, a lysis zone without secondary colonies was observed; accordingly, it was marked as “++++”. On day 7 after hydrogel formation, lysis zones were noted in all culture dishes, but they did not extend beyond the contours of the phage-containing samples. In sample 4, containing bacteriophages, succinic acid, and lidocaine, secondary colonies were observed in both experimental series, i.e. the lytic activity there was graded as “+++”, and in samples 1, 2, 3 as “++++”.

## Discussion

Due to the wide-spread multidrug resistance of microorganisms, the therapeutic use of bacteriophages has attracted a lot of attention from researchers and practitioners [1]. A number of research groups in Europe, Asia, and the United States are focusing on creating and maintaining a sufficient concentration of bacteriophages in infection-susceptible skin wounds. The use of hydrogels to create a depot of bacteriophages is considered a promising direction. For example, in a joint study of French, Belgian, and Swiss scientists on the novel product “PhagoBurn” [3], it was concluded that a stable therapeutic concentration of bacteriophages was crucial for a successful phage therapy. Due to the extremely small (up to 100 nm) sizes of viral phages, maintaining their therapeutic efficacy in local preparations is a complex technical problem. Therefore, it was proposed to immobilize phages in the structure of polymeric carriers in order to prolong the period of their activity within the wound dressing [10]. In addition, combined drug therapy aimed at multiple targets improves the treatment efficacy and reduces adverse events [11]. Among other measures, using multicomponent dressings for soft tissue wounds is widely discussed in the medical literature. In order to ensure the optimal wound healing, the dressing material must be biocompatible, biodegradable, and porous so to resemble the structure of normal skin. In this regard, one of the most promising candidates is a dressing based on a hydrogel [12]; these gels have a three-dimensional hydrophilic polymer network capable of absorbing large amounts of water and biological fluids [13]. Due to this property, hydrogels can mimic natural living tissues more than other synthetic biomaterials. The unique physical properties of hydrogels rationalize their use for the immobilization and subsequent gradual release of drugs in the area of local application. The physicochemical properties of hydrogels make it possible to load drugs into the matrix and then release them through the gel network at a rate that depends on the diffusion coefficient of a particular molecule [14]. The rate and duration of drug release are regulated by passive diffusion mechanisms and depend on factors such as the size of the network and the capacity of the hydrogel [15]. Earlier, the possibility of maintaining the viability of bacteriophages for 21 days in a gel based on calcium alginate was demonstrated [16]. In other reports, chitosan [17] and PVA [18] were used to prepare hydrogel wound dressings. For the present study, PVA — a biocompatible and non-toxic polymer — was chosen [19].

The results obtained in this study indicate that the proposed hydrogel itself does not possess any lytic activity against microorganisms. However, when saturated with a solution of bacteriophages, the hydrogel-based wound dressing expresses an antibacterial activity that persists for 7 days.

Of particular, interest is the possibility of saturating a polymer film (which is a base for a hydrogel wound dressing) with a solution of phages that are active against bacterial pathogens specific for a given medical facility. This would enable the local doctors to respond timely to the appearance of antibiotic-resistant bacteria in the zone of surgical intervention.

Equally important is the possibility of combining bacteriophages with anti-hypoxic agents and anesthetics. Succinic acid is not only a substrate necessary for tissue respiration, but it is also a metabolic trigger of adaptive mechanisms needed for wound healing [20]. The present study showed that the addition of succinic acid into the hydrogel wound dressing reduced the lytic activity of bacteriophages by day 7 to the “+++” level. However, given the fact that in clinical practice, a dressing is applied on the wound for 4–5 days only, the result obtained is clinically significant.

## Conclusion

The structural properties of hydrogels and their affinity for certain bioactive molecules can play a key role in creating a system for delivery of therapeutic agents to the area of surgical intervention. Specifically, hydrogels provide the possibility of immobilizing bacteriophages and combine them with succinate and lidocaine for the antibacterial treatment of surgical wounds.
